# Artificial neural networks for diagnosis and survival prediction in colon cancer

**DOI:** 10.1186/1476-4598-4-29

**Published:** 2005-08-06

**Authors:** Farid E Ahmed

**Affiliations:** 1 Department of Radiation Oncology, Leo W Jenkins Cancer Center, The Brody School of Medicine at East Carolina University, Greenville, NC 27858, USA

**Keywords:** ANN, backpropagation, nodes, perceptron, performance, training, weights

## Abstract

ANNs are nonlinear regression computational devices that have been used for over 45 years in classification and survival prediction in several biomedical systems, including colon cancer. Described in this article is the theory behind the three-layer free forward artificial neural networks with backpropagation error, which is widely used in biomedical fields, and a methodological approach to its application for cancer research, as exemplified by colon cancer. Review of the literature shows that applications of these networks have improved the accuracy of colon cancer classification and survival prediction when compared to other statistical or clinicopathological methods. Accuracy, however, must be exercised when designing, using and publishing biomedical results employing machine-learning devices such as ANNs in worldwide literature in order to enhance confidence in the quality and reliability of reported data.

## 1 Introduction and Development of Artificial Neural Networks

Artificial neural networks (ANNs) are regression devices containing layers of computing nodes (crudely analogous to the mammalian biological neurons) with remarkable information processing characteristics. They are able to detect nonlinearities that are not explicitly formulated as inputs, making them capable of learning and adaptability. They possess high parallelism, robustness, generalization and noise tolerance, which make them capable of clustering, function approximation, forecasting and association, and performing massively parallel multifactorial analyses for modeling complex patterns, where there is little *a priori *knowledge [1]. Artificial neural models possessing such characteristics are desirable because: (a) nonlinearity allows better fit to the data, (b) noise-insensitivity leads to accurate prediction in the presence of uncertain data and measurement errors, (c) high parallelism implies fast processing and hardware failure-tolerance, (d) learning and adaptability permits the system to update and/or modify its internal structure in response to changing environment, and (e) generalization enables application of the model to unlearned data [2].

**Figure 1:**
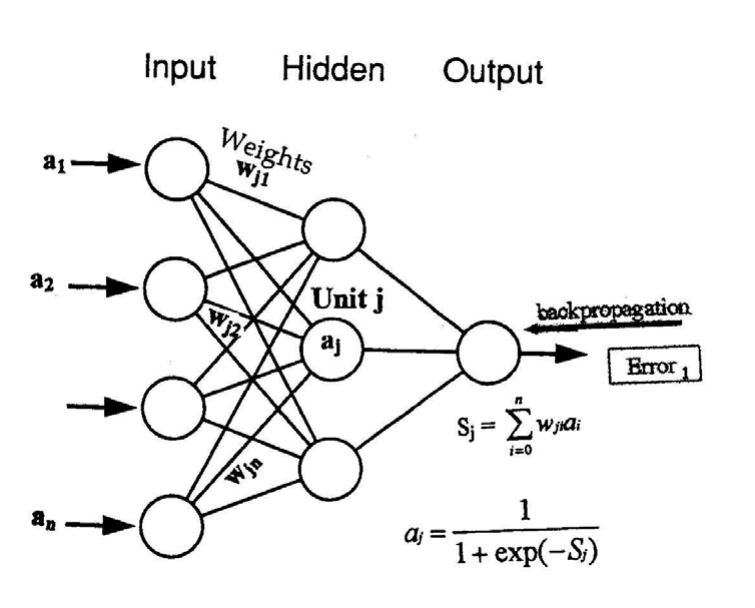
A three fully interconnected feedforward BP neural network (FFNN), with a single hidden layer. From reference 2, with permission.

**Figure 2:**
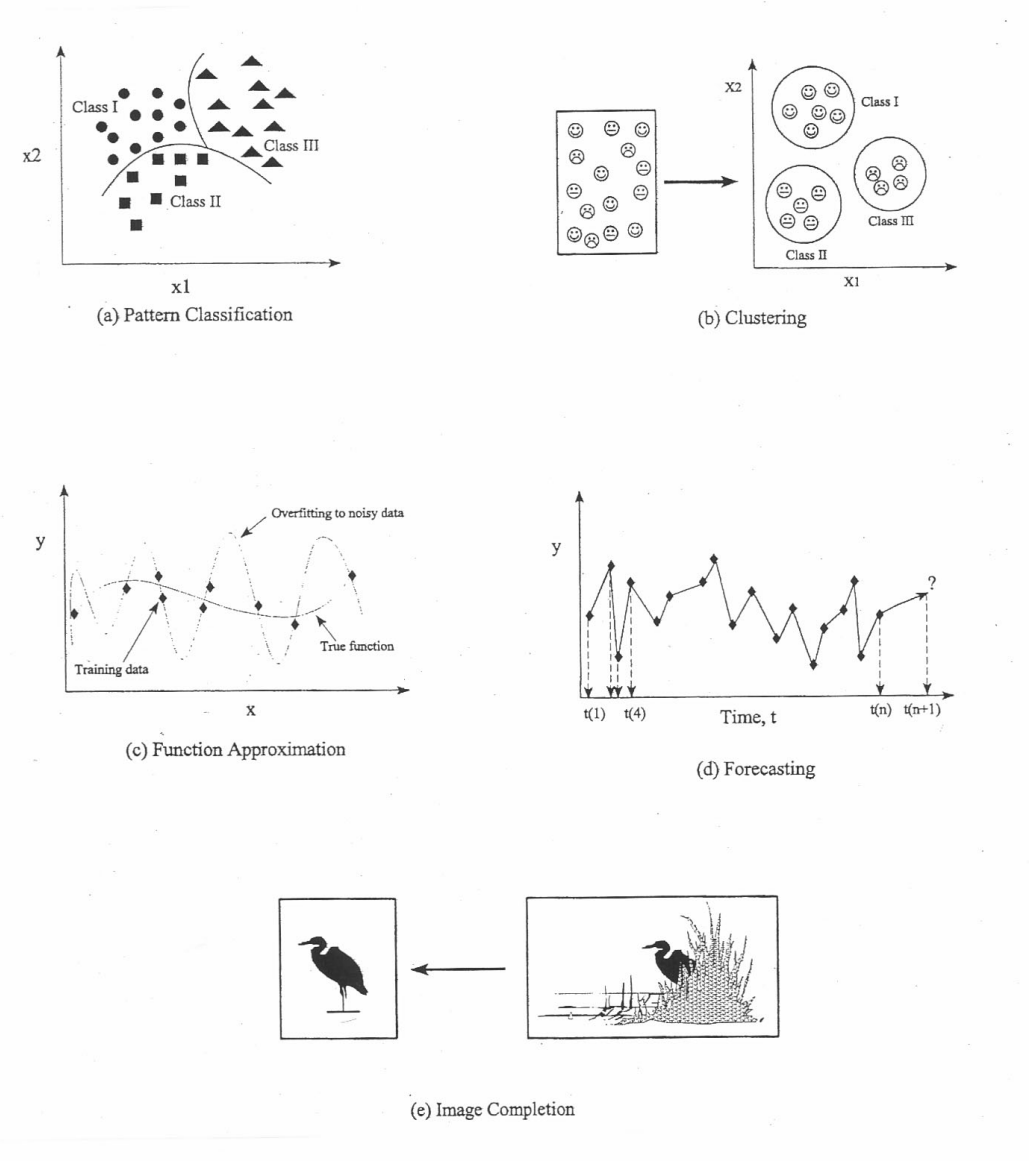
Application of ANNs to problem solving: (**a**) pattern classification (i.e., assigning an unknown input pattern to any of prespecified classes based on properties that are characteristic to a given class); (**b**) clustering (i.e., clusters or classes are formed by exploring the similarities or dissimilarities between the input patterns based on their inter-correlations); (**c**) functional approximation or modeling (i.e., training an ANN on input-output data to approximate the underlying rule relating the inputs to outputs); (**d**) forecasting or predicting (i.e., training an ANN on samples for a time series [t(1) to t(n)] representing a certain phenomenon at a given scenario and then using it for other scenarios to predict the behavior at a subsequent time [t(n + 1)], and (**e**) association (i.e., developing a pattern by training an ANN to construct the corrupted or missing data).

In the early 1940s, McCulloch and Pitts [3] explored the competitive abilities of networks made up of theoretical mathematical models when applied to the operation of simple artificial neurons. When these early neurons were combined, it was possible to construct networks capable of computing any of the finite basic Boolean logical functions, including symbolic logic. The system comprised of an artificial neuron and input (stimuli) was referred to as "the Perceptron", which established a mapping between input activity and output signal. The next important milestone was the development of the first trainable network perceptron by Rosenblatt, 1959 [4] and Widrow & Hoff, 1960 [5], initially as a linear model having two layers of neurons or nodes (an input and an output layer) and a single layer of interconnections with variables (weights) that were adjustable during training. Some models increased their computational capabilities by adding additional optical filters and layers with fixed random weights, or other layers with unchanging weights. However, these single layers of trainable weights were limited to only solving linear problems. By 1974, Werbos [6] expanded the network to have nonlinear capabilities, modeling with two layers of weights that were trainable in a general fashion, and that accomplished nonlinear discrimination and functional approximation. These original algorithms were named "back-error propagation, BP" and the networks called multilayer perceptrons (MLPs). In BP, the network error (i.e., difference between the predicted and true outcome) constitutes two steps: forward activation to produce a solution, and a backward propagation of the computed error to modify the weights (usually carried out through fitting the weights of the model by a certain function, such as squared error or maximum likelihood, using a gradient optimization method) [Figure 1]. Rumelhart and McClelland popularized ANNs in 1986 [7], and a variety of ANN paradigms have been developed over the last 46 years [2]. In fact, over 50 different ANN types exist. Some applications may be solved using different ANN types, whereas others may only be solved by a specific ANN type. Some networks are capable of solving perceptual problems, while others are more tailored for data modeling and functional approximation [8]. Within cancer research alone, ANNs have been applied to image processing, outcome prediction, treatment-response forecasting, diagnosis and staging [1] [Figure 2].

After demonstrating the utility of ANNs to various applied problems, mathematicians established a theoretical basis for the conceptual capabilities of the MLPs. They showed by a general function approximation theorem that, with appropriate internal parameters (or weights), a neural network could approximate an arbitrary nonlinear function [2]. Thus, ANNs should not be viewed as "black boxes", but as tools that are capable of learning and outcome prediction. Due to the fact that classification tasks, prediction issues and decision support problems are considered functional approximation problems, then ANNs could be applied to problem solving in various domains, and a major research effort has been dedicated to ways of adjusting weights to the best-fitting functional approximations and training parameters [8].

When an ANN is trained on a set of data, it builds a predictive model that reflects a minimization in error when the network's prediction (its output) is compared with a known or expected outcome. Training, which is analogous to biological learning, is carried by a "teacher" program that loads in training cases from a database and adjusts the weights and thresholds value of the network to minimize the error between the real-world outputs and the network generated outputs for the training case inputs. The network would then be validated with available data, and performance measurements [e.g., the mean squared error (MSE), the full range of sensitivity and specificity values (i.e., receiver operating characteristic, ROC, plot associated with the continuous variable output, 0 to 1), and confidence and prediction intervals] can ascertain the network's level of success in arriving at a meaningful prediction unique to each input. Traditionally in medicine, expert opinions have been developed from clinicians' experience and search of the literature. Today, however, ANNs and multivariate analysis can be used to analyze the multitude of data simultaneously and to learn tends in population, thus expanding the "localized" knowledge to a more "global" knowledge, which can be accessed by other practitioners [8].

## 2 Theory and Performance Measures Behind the Feedforward Artificial Neural Netwoks (FFANN)

The feedforward BP MLP can be viewed basically as a set of equations that are linked together through shared variables in a formation diagramed as a set of interconnected nodes in a network capable of general functional approximation that provides learning capabilities [9]. Variables for inclusion in the final network architecture are usually chosen by a sensitivity analysis method, which tests each input variable by dropping it from the input list and determining the resulting loss of predictive accuracy. Only variables that result in a significant loss of accuracy when dropped are retained in the final network's architecture. Classification tasks like tumor staging, diagnosis, or predicting survival can be performed by FFANNs [10].

**Figure 3:**
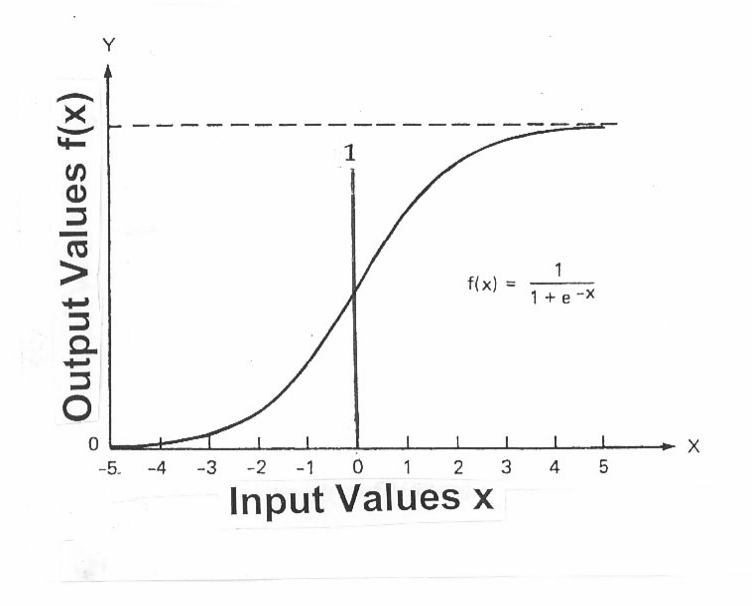
The activation, squashing, or sigmoid function f(x).

FFANN is typically organized as a set of interconnected layers of artificial intermediate (hidden) nodes depicted as a row or collection of nodes, each receiving input from other nodes, connected together to form the network (Figure 1). The MLP has an associated output activation level known as a "squashing" or "activation" function; the most popular is the sigmoid function [f(x)] expressed as:

f(x) = 1/[1 + exp(-x)] ..................................... (**1**), where x is the input to the squashing function (Figure 3). This sigmoid function, which may have no biological significance, can then be expressed as S_j _(the sum of the products of the incoming activation levels with their associated weights):

, where W_ji_, is the incoming weight for unit i; a_i_, is activation value of unit i; and n, is the number of units that send connections to unit j.

**Figure 4:**
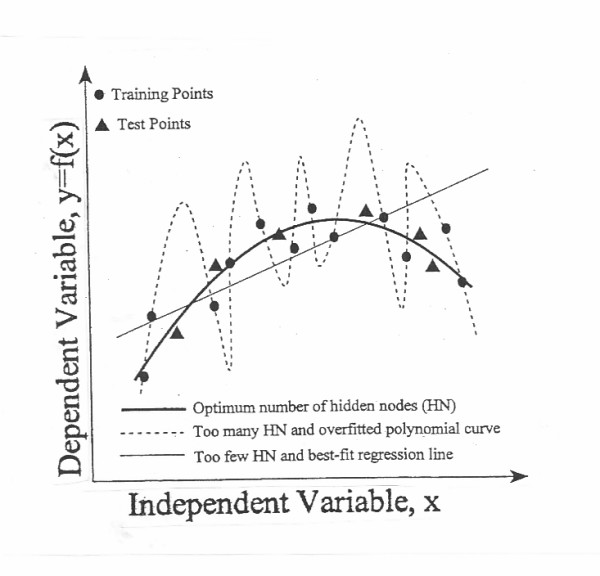
Effect of hidden layer size on network generalization. From reference 2; with permission.

The majority of biomedical studies utilize three-layer networks (input, intermediate and output), in which layers are fully connected (Figure 1). Each connection has an associated weight (w) that corresponds to synaptic junctions in biological systems. Equation (1) becomes:

, where a_i, k _represents the activation value of node 1 in layer k, and W_ji, k _represents the weight associated with the connection from the ith node of the kth layer to the jth node of layer k + 1. In a three layer node ANN, there exist two types of weights, and k = 1 or 2. Whereas a network with too few hidden nodes would be incapable of differentiating complex patterns, a network with too many hidden nodes lead to poor generalization for untrained data (Figure 4) [2].

The most popular approach to finding the optimal number of hidden nodes (HN) is by trial and error. Other statistical methods such as cross validation, bootstrapping or pruning have been used. Livingstone & Manallack's [11] suggested that hidden node can be empirically expressed as: HN = M·O/W ..................................... (**4**), where M is the number of training examples; O, is the number of outputs; and W, is the number of weights, and HN is usually >3 to ensure good generalization and avoid memorizing the training set. Thus, if there are 240 training cases and a single output, the network should not have more than 80 weights. In a network with 10 inputs, this corresponds to having a single hidden layer with 6 units [12].

In theory, there are some problems for which it may be better to use a network with two hidden layers because the overall number of nodes will be less than it would be in a single-hidden layer net. However, for most biomedical applications there is no substantial practical evidence that more than one hidden layer will add meaningfully to the predictive capabilities of a network. Therefore, for practicality, most medical applications use a single hidden-layer networks [12].

In an untrained ANN, the weights of all interconnections are set to be small random numbers. The ANN is then trained (i.e., presented with a training data set that provides inputs and desired outputs of the network). The weights are continuously adjusted by algorithms such as gradient descent computation *et sequa*, that seek to find a minimum in the error surface so that the network computes the desired output [13]. The amount of network error (or mean square error, MSE) is expressed as:

, where d_i, p _is the desired output of output unit i for input pattern p; P, is the total number of patterns in the data set; n, is the number of output units, and the sum is taken over all data patterns and all output units. The root mean square (RMS) is the square root of the MSE.

Gradient descent weigh training starts with inputting a data pattern to the network in order to determine the activation values of the input nodes. This is followed by forward propagation, in which the hidden layer updates its activation value followed by updates to the output layer (as depicted in equation **3**). Next, the desired (known) outputs are submitted to the network. A calculation is then carried out to assign a value to the amount of error associated with each output node. The formula for this error value (δ) is expressed as:

δ_j, 3 _= (d_j _- a_j, 3_) f(x) (S_j, 3_) ..................................... (**6**), where d_j_, is the desired output for output unit j; a_j, 3_, is the actual output for output unit j (layer 3); f(x), is the squashing function; and S_j, 3 _is the incoming sum for output unit j in equation (**2**).

**Figure 5:**
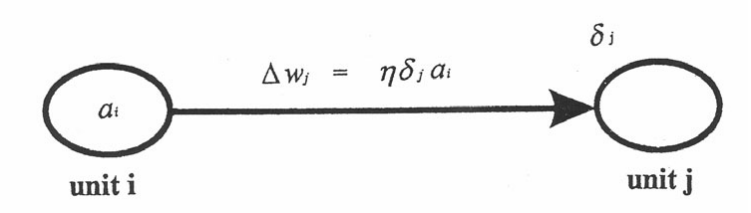
Updating or adjusting the value of the weight along a single connection. From reference 8; with permission.

After these error values become known, weights (from unit i to j) on the incoming connections to each output neuron can be updated according to the following equation:

ΔW_ji, k _= ηδ_j, k+1 _a_i, k _..................................... (**7**), in which k = 2 during updating the layer of weights on connections that terminate at the output layer (see Figure 5).

As the BP ensues, an error value (δ) is then calculated for each hidden node as follows:

δ_i, 2 _= (∑δ_j, 3 _W_ji, 2_) f(x) (S_i, 2_) ..................................... (**8**).

After the error values are known, weights on the incoming connections to each hidden neuron can then be updated. The updated equation (# **7**) is used again, substituting k = 1 for weights on connections that start at the first layer. The derivation of the above equations is based on the gradient descent approach and uses the chain rule and interconnected structure of the network [6,8,13]. A general function approximation theorem has been proven for a three layer MLP, showing that they are capable of approximating any nonlinear function in such a way that creating the functional form and fitting the function are performed at the same time (Figure 5), unlike nonlinear regression in which a fit is forced to a prechosen function, giving the ANN an advantage over traditional statistical multivariate regression techniques [14].

**Figure 6:**
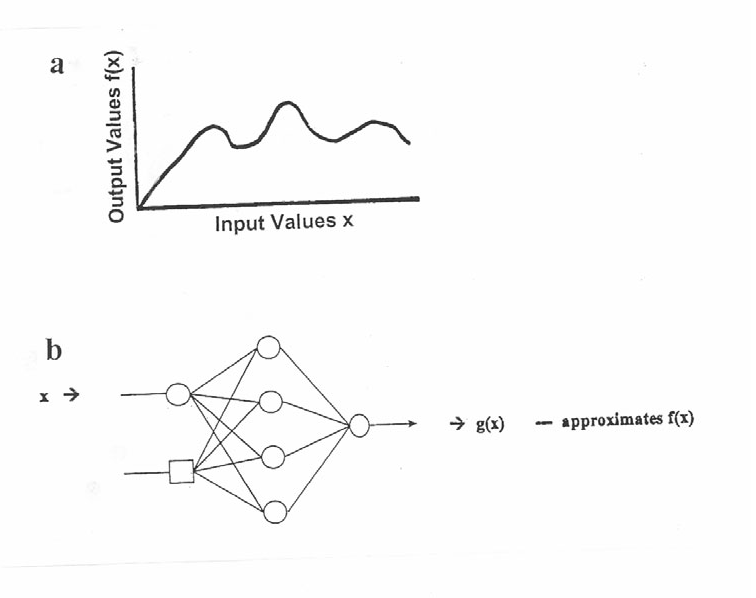
Example of a nonlinear functional approximation configured by ANN weights. (**a**) Illustration showing a function f(x). (**b**) A neural network configuration to determine an approximation to f(x), given the input x. Modiefied from reference 8.

Figure 6 is a graphical representation of an arbitrary nonlinear function approximation performed by an ANN. The function computes a value y = f(x) for every value of x. The ANN is trained to input the value of x, and to output an approximation of f(x). ANN weights are available, which are capable of approximating any arbitrary nonlinear function [8].

**Figure 7:**
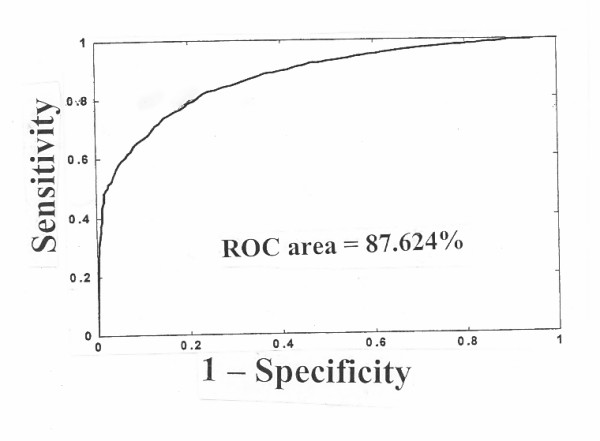
ROC curve for validating cases from an ANN. Modified from reference 10.

It should be noted that there has to be patterns (or predictive factors) present in the training data for the ANN to learn successfully; otherwise the network's performance will be low. To measure how well a single output ANN matches data with known outcome, performance metrics include the MSE and RMS. The area under the ROC, or AUROCC, [in which sensitivity can be blotted as a function of (1 - specificity)] (Figure 7) is an acceptable performance measure to use with a single output classification neural network [15]. AUROCC gives a definitive measure of the classifier's discrimination ability that is not dependent upon the choice of the decision threshold. It is identical to the probability that given a positive case and a negative case, the network output will be higher for the positive case. Algorithms are available for calculating the AUROCC [15]. Although the AUROCC provides a useful measure of discrimination (i.e., how well a prediction model can rank patients), it does not, however, provide much insight into calibration (which refers to the correspondence between predicted and actual probabilities). Calibration curves, which are plots of actual against predicted probabilities, are very useful for visually determining accuracy, and can generally help a physician make better inferences before he provides a predicted probability to a patient under evaluation [16].

Other measures of the network's performance include the kappa value and the information given. Unlike the AUROCC, these measures require an output threshold to be chosen. Kappa is the actual improvement in classification rate over the chance rate divided by the maximum possible improvement over the chance rate. A value of 1 indicates perfect classification, and a value of 0 indicates classification at the chance rate [12].

The information gain refers to the decrease in classification uncertainty after having observed the network output. Algorithms are available for finding the output threshold that maximizes the information gain [17]. If the relative costs of different types of misclassification (i.e., the cost of false negative or false positive) are known, then an overall cost measure can be calculated. Alternatively, the network can be tuned to output these values directly [12].

**Figure 8:**
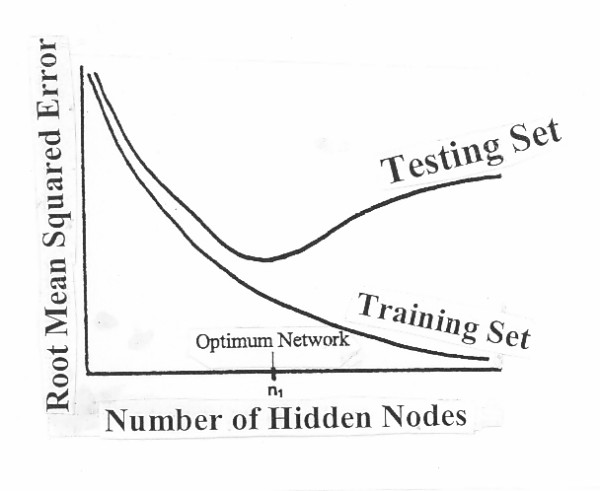
Criteria for termination of training and selection of an optimum ANN architecture. Modified from references 2 and 8.

When training an ANN, three non-overlapping sets of data are used: (a) the training set, (b) the validation (or testing) set, and (c) the verification set. The training set is used for adjustment of weights during training, whereas the testing set is used to decide when to stop training; otherwise, the ANN will learn features in the training set that are not present in the wider population of cases, a phenomenon known as "fitting to noise" or "overfitting". The performance measures should be made on both the training and test sets. However, only if the testing set has been used to set the network's weights or evaluate its structure, will it reflect the network's performance on future data; this practice of splitting the data into a training set and a test set is referred to as "cross validation" [18]. Another method for estimating the error rate of a prediction rule is "data splitting" [19]. Both cross validation and data splitting methods are suitable if there is plenty of data available. For small data sets, the "bootstrap" method is used. A drawback of the bootstrap is that a large number of samples (20 ≤ B samples ≤ networks) must be trained [20]. As seen in a classical training curve (Figure 8) where RMS of the training and testing sets are plotted as a function of the number of HNs or training cycles, the RMS on the training set decreases as more training is carried, but the testing set has a minimum when reached the RMS begins to increase. Training beyond the inflection point results in overfitting. It is imperative to not overfit the network during training, which can be achieved by methods such as restricting the topology of the network (i.e., decreasing the number of nodes), or early stopping, or by using weight decay. If computationally possible, one should consider the use of a Bayesian approach that averages over several plausible networks [21].

The time it takes to train a neural net increases exponentially with the number of network inputs and the number of network nodes, and polynomially with the number of training examples. A network with 200 inputs trained on a few thousand examples takes about four hours to train on a computer. Therefore, it is important to include only those inputs and examples that seem relevant to the task at hand. This is not a significant problem for medical decision-making, as they are generally small. For example, a typical medical net has 20 inputs and is generally trained on ~350 samples [12]. An ANN-based system is considered to have learned if it can: (a) handle imprecise, fuzzy, noisy and probabilistic information without noticeable adverse effect on response quality, and (b) generalize from the tasks it has learned to an unknown ones [2].

It should be remembered that because the ANN has undergone generalized learning, it becomes capable of interpolating or extrapolating results from new incoming data. However, it is important to ascertain that the incoming data do not extend beyond the range of values used in training. Data outside acceptable limits should either not be processed or results flagged and viewed with caution. For data within the range of network training, a measure of confidence can be made by calculation of a confidence or a prediction interval. It should be kept in mind that a trained, tested and verified ANN does not provide a unique solution because its final trained resting state depends on several factors such as number of original randomized starting weights, number of and order of presentation of cases, and the number of testing cycles. Other mathematical alternatives employed during training such as the use of momentum, adjusting the learning constant, "jogging" the weights, etc., may have implications. Therefore, for a particular application such as cancer prediction, a frequency distribution of the network versus the outcome probability can be produced and a central tendency such as mean, mode, measure of variance and nonparametric predictive intervals (in case of skewed nonparametric distributions) are plotted, producing what is called "prevalence-value accuracy" plots. Then it could be stated, for example, that with 90% confidence, the probability of the expected outcome will occur with such a range, having a median value of such a number [22].

Rather than putting faith in "black box" systems, the workings of a neural net set used in survival analysis on censored data could be explained by exploring the interactions between predictive values and survival rates, which leads to useful insights into the roles played by different prognostic variables in determining patient outcomes [23]. In a performance measure approach dubbed a "sensitivity analysis", each input is varied and the corresponding change in output is measured. The ratio of change in output over input (δ_y_/δ_xi_) is then averaged over all samples to produce a sensitivity parameter for each input. The inputs can then be ranked according to sensitivity. Sensitivity analysis, however, could be misleading because y may not be a linear function of x. The sensitivity measures are dependent on the particular example used to train the network, and also on the initial weight setting of the network [12].

Another performance measure approach known as "key factor justification" has been used to explain individual decisions. In that approach, each input to the ANN is reversed. If the output decision is consequently reversed, then a key factor could accordingly be identified. If no single key factor could be ascertained, then pairs of variables, or triples, could be reversed together and the output is observed. However, going beyond triples may require excessive computational capabilities [24].

## 3 General Application and Improving Performance of ANNs

ANNs have been applied to problem solving in various fields including: (a) pattern classification, (b) clustering (class separation), (c) functional approximation (modeling), (d) forecasting, (e) association (e.g., image completion), and (f) optimization (e.g., finding a solution that minimizes an objective function (see Figure 2) [2].

In the military and electronic arenas, ANN applications include automatic target recognition, control of flying aircrafts, engine combustion optimization, adaptive switching, circuits and fault detection in complex systems [8]. In the financial field, a decision support role for ANNS to predict stock market fluctuations and commodity trading has been envisioned [8]. In the biological domain, ANN application to samples' characterization, identification and interaction include: interpreting pyrolysis mass spectrometery, GC and HPLC data; pattern recognition of DNA, RNA, protein structure and microscopic images; prediction of microbial growth, biomass and shelf-life of food products; and identification of microorganisms and molecules [2]. In the medical and behavioral sciences, image analysis has resulted in systems capable of diagnosis and prognosis of various diseases, (including cancer), classification of cancer subtypes, predicting tumor sensitivity to drugs, identification of potential biomarkers, analysis of gene expression data, medical imaging and radiological diagnosis, analysis of wave forms, outcome prediction, identification of pathological specimens, interpretation of laboratory data, evaluation of epidemiologic data, waveform analysis (including electroencephalography, electromyogram, electrocardiogram and Doppler ultrasound), length of stay in intensive care units following various diseases/surgery, and predicting admission decisions in psychiatric wards [25-30].

The performance of an ANN depends on network parameters, the network weights and the type of transfer functions used. A disadvantage of using FFANNs is that they require the initialization and adjustment of many individual parameters to optimize their classification performance. When optimized manually, these adjustments can take days or even weeks to complete one set of experiments for estimating one outcome on single database [31]. The lengthy process of manually optimizing a feedforward BP ANN provided the incentive to develop an automated system that could fine-tune the network parameters without user supervision. A new stopping criterion (i.e., the logarithmic sensitivity index) was introduced that provided a balance between sensitivity and specificity of the output classification. The network automatically monitored the classification performance to determine when was the best time to stop training after no noticeable improvement in the performance measure (either highest correct classification rate, lowest mean squared error, or highest log-sensitivity index value) occurred in the subsequent 500 epochs. Using these automated ANNs, experiments performed on three medical databases showed that the optimal network parameter settings found by the automated system were similar to those found manually, and that automated networks performed equally well or better than the manually optimized ANNs, and the best classification performance was achieved using the log-sensitivity index as a stopping criterion [31].

When using an ANN, three important issues needs to be addressed that the solution to which will significantly influence the overall performance of the ANN with regard to two considerations: (a) recognition rate to new patterns, and (b) generalization performance to new data sets that have not been presented during network training [32]. These issues are: (i) the selection of data patterns for network training [33], (ii) the selection of an appropriate and efficient training algorithm from a large number of possible training algorithms found in the literature such as BP and its many variants [34] and the second-order algorithms [35], just to name a few. New training algorithms with faster convergence properties and less computational requirements are being developed, and (iii) determination of network size. This is a more difficult problem to solve. It is necessary to find a network structure small enough to meet certain performance specifications. In practice, this is carried by training a number of networks with different sizes, and the smallest network that can fulfill all or most of the required performance requirements is selected. In an attempt to develop a systematic procedure for an automatic determination and/or adaptation of the network architecture to an incremental constructive training scheme, input-side and output-side training could be separated in order to improve the input-side training effectiveness and efficiency, and to obtain better generalization performance capabilities. Two pruning methods for improving the input-side redundant connections were also developed that resulted in smaller networks without degrading or compromising their performance. Moreover, numerical simulations demonstrated the potential and advantages of the proposed data pattern selection/training and size determination strategies when compared to other existing techniques in the literature [32].

## 4 Applications of ANNs to Colon Cancer Diagnosis

Microarray data are becoming powerful tools in clinical diagnosis, particularly for tumor classification because they simultaneously record gene expression levels of thousands of genes. These data are characterized by high dimensionality because a large number of gene expression input vastly exceeds the number of sampling, which may lead to overfitting. This situation necessitates dimensionality reduction through either using a reduction algorithm, or selecting a small set of genes as input to the classifier in a supervised way [36], or by employing cross validation to avoid overfitting [37].

Both unsupervised clustering methods and supervised methods have been used for classification [38]. I have employed colon cancer as an example to show how supervised ANNs have an advantage over clustering methods (which were shown to be incapable of detecting subtle differences between biological classes) in classification if some prior knowledge of the classes is available.

There is an important subtle distinction between sporadic colon adenomas and cancers (SACs) and inflammatory bowl disease-related dysplasia or cancer (IBDNs) because SACs can be managed by polypectomy alone, whereas IBDNs require a life-threatening subtotal colectomy. A microarray study was conducted to evaluate the ability of ANN and hierarchical cluster analysis to discriminate between these types of cancer based on hybridizing 8064 cDNA clones to mRNAs derived from 39 colon neoplastic specimens [1]. GeneFinder software was used to select 1192 clones that showed significantly different mean square expression levels between IBDNs and SACs (P = 0.001). A BP FFNN, with two hidden layers and 1192 inputs (representing the selected genes) was constructed, and the output was set at 0 for IBDNs and 1 for SACs using the software program MatLab (Math Works, Inc., Nattick, MA). The ANN was learned using a training set of 5 IBDNs and 22 SACs. The test set comprised the remaining data samples consisting of 3 IBDNs and 9 SACs. ANN approximations were evaluated using regression analysis that compared expected output (Target) with ANN output following training, and unpaired 2-sided Student t-test was also used to evaluate the statistical differences between the net defined IBDNs versus SACs (i.e, 0 vs. 1). Hierarchial clustering was performed using the program Cluster (Stanford University, Palo Alto, CA). Whereas the network correctly diagnosed 12 of 12-blinded samples, hierarchial analysis failed, probably because of noise in the database. Only by using an iterative process to reduce the number of clones used for diagnosis to 97, could cluster analysis separate the two types of lesions. Even with this reduced clone set, ANN still retained its capacity for correct diagnosis of the two types of colon cancer [1].

Another microarray study employed a combination selection method in conjunction with ensemble neural network to analyze cancer data, including that of the colon. The principle of the method was based on the assumption that combining various feature selection mechanisms to chose top-ranked genes will avail more information, and by using an ensemble combining the output of several ANNs into an aggregate output, features can be analyzed more effectively due to the stability of the networks and robustness of the answers [39]. The authors employed the public database of Alon et al [40] containing 62 samples (40 colon tumors and 22 normal tissue samples). They chose 2,000, out of ~6,500 expressed genes, based on their confidence in the measured expression level to assemble networks consisting of 100 members. No fresh samples were available for testing the network ensemble. Nevertheless, using this ensemble, the predictive accuracy of adopting leave-one-out cross validation (LOOCV) and 10-fold cross validation was 91.94% and 90.32%, respectively, as compared to 85.48% obtained by using various boosting algorithms in combination with LOOCV. However, a drawback of the ANNs ensemble approach is the increased computational complexity and the additional time needed to perform the analysis [39].

## 5 Application of FFNN to Predicting Survival in Colon Cancer

It is currently difficult to predict when and if a particular patient will die after surgical and adjuvant chemotherapeutic treatment of colon cancer, especially at the intermediate Dukes; B and C stages, using available techniques based on histopathological TNM staging and employing univariate and multivariate regression analysis [41].

A 5-year follow-up data from 334 patients treated for colorectal cancer (CRC) were used to train 284 patients and validate 50 patients using 6 FFNN with BP, containing from 2 to 15 hidden units designed to predict death within 9, 12, 15, 18, 21 and 24 months using the logistic activation function with continuous output on the interval 0, 1. Furthermore, the trained 12-months ANN was then applied to 2-years follow up on patients from a second institution. The network predictions of which individual patients would die within 12 months were also compared with those of two consulting surgeons [42]. Results showed that all 6 ANNs were able to achieve an overall predictive accuracy of death at 95% CI ≥ 80% at the first institution, with a mean sensitivity and specificity of 60% and 88%, respectively. Furthermore, the trained 12-months ANN achieved an overall predictive accuracy for death of 90% (95% CI 84–96) when applied to death from the second institution, compared with an overall accuracy of 79% (71 – 87) and 75% (66 – 84) for CRC surgeons. Thus, ANNs predicted outcome for CRC death more accurately than clinicopathological methods. Moreover, once trained in one institution, ANNs were able to accurately predict outcome for patients from an unrelated institution [42].

In another study to predict a 5-year survival after primary treatment of colon carcinoma in the National Cancer Data Base (NCDB), UK, 37,500 cases treated between the years 1985 and 1993, and not used in model development, were analyzed by an ANN model and compared with a standard Cox parametric logistic regression [10]. A FFNN with two hidden layers that contained 4 and 3 hidden neurons, respectively, and one output layer was selected. Eleven input variables were chosen by a sensitivity analysis method (including race; sex; age; tumor location, size, behavior; histopathology; surgery, chemo or radiation therapy, hormonal or other cancer-directed therapy) and only the variables that resulted in significant loss of accuracy when dropped were retained in the final network architecture, Training of the network was accomplished by using a standard second order conjugate gradient descent method. A validation set representing 25% of randomly chosen data was employed for validation. The area under the ROC curve was used to measure the overall predictive accuracy of the network. The ANN yielded a ROC area of 87.6%. At sensitivity to mortality of 95%, the specificity was 41%. The logistic regression yielded a ROC area of 82%, and sensitivity to mortality of 95% gave a specificity of only 27%. Thus, the ANN found a strong pattern in the database predictive of 5-year survival status, whereas the logistic regression produced somewhat less accurate, but good results [10]. In another study by the same group of investigators [43] aiming at predicting 5-year survival associated with CRC using the same ANN and Cox regression and ROC to compare data, the logistic regression model gave a result of 66% and the ANN gave 78%, indicating that the neural network approach was more superior compared to regression analysis in predicting colon cancer survival.

A fourth study compared ANNs to TNM staging to predict 5-year survival of patients with CRC, using the area under the ROC as a measure of accuracy. Variables for patient care evaluation (PCE) database used for analysis included: age, race, gender, signs and symptoms (e.g., changes in bowel habits, obstruction, jaundice, occult blood, and others), diagnostic and extent-of-disease tests (e.g., endoscopy, radiography, barium enema, colonoscopy, CT, biopsy, CEA antigen, X-ray, liver function tests and others), and histoipathological parameters. A test set of 5,007 training cases, and a validating set of 3,005 cases was used. A FFNN BP composed of an input, a hidden and an output layer was used. The ANNs prediction of 5-year survival was significantly more accurate than the TNM staging (ANN 0.815 versus TNM 0.737, p < 0.001). Adding commonly collected demographic and anatomic variable to the TNM variables further increased the accuracy of the ANN (0.869). Thus, the ANNs were significantly more accurate than the TNM staging system when both used the TNM prognostic factors alone, and prognostic factors added to ANN further increased the predictive prognostic accuracy [44].

## 6 Conclusion

There are advantages and disadvantages to FFANNs when applied to biomedical decision-making. Advantages include: (a) requirement for less formal statistical training to develop, (b) having a better discriminating power than other regression models, (c) can be developed using multiple different training algorithms, (d) their parallel nature enable them to accept a certain amount of inaccurate data without a serious effect on predictive accuracy (i.e., graceful degradation), (e) having the ability to accurately detect complex nonlinear relationships between independent and dependent variables, and all possible interactions between variables, as they make no assumptions about those variables, (f) reduce the number of false positives without significantly increasing the number of false negatives, and (g) they may allow for individual case prediction. On the other hand, disadvantages include: (a) considered as "black box" methods, one cannot exactly understand what interactions are being modeled in their hidden layers as compared to "white box" statistical models, (b) have limited abilities to identify possible causal relationships, (c) model development is empirical; thus, providing low decision insight, and many methodological issues remain to be solved, (d) models prone to overfitting, (e) require lengthy development and time to optimize, (f) they are more difficult to use in the field because of computational requirements, and (g) there is conflicting evidence as to whether or not they are better than traditional regression statistical models for either data classification, or for predicting outcome [21,45,46].

Despite their theoretical advantages, ANNs do not universally outperform standard regression techniques for several reasons: (a) because from a practical point of view, only a limited amount of data that may be related to the outcome of interest can be collected, and these data are mostly based on studies in which a standard regression model was used, and therefore only factors that were significant in a regression models are collected in subsequent studies. Therefore, nonlinear functions, or those that involve interaction with other variables may not have emerged as "significant" in the regression analysis and therefore are not reflected in the literature as important prognostic factors, (b) all variables and outcomes are measured with error(s). A nonlinear relation when measured with an error may well be adequately represented by a linear model, (c) there exist data barriers beyond which mathematical models are unable to make predictions in biological systems, and (d) regression models are superior to ANNs when drawing inferences and interpretations based on outputs [47]. In addition to insight into the disease process, regression models provide explicit information regarding the relative importance of each independent variable. This information can be valuable in planning subsequent interventions, in eliminating unnecessary tests or procedures that are unrelated to the outcome of interest, and in determining which are the most critical data to store in the database [47].

Although the representation of a complex risk structure by nonlinear machine-learning methods such as ANN or classification and regression tree (CART) could provide as insight into the underlying nature of a disease, ease of interpretation is not a typical feature of the network representation of a complex relationship. However, a suitable network approach could outperform other approaches, provided that the underlying disease has sufficient complex interactions because the ability to represent arbitrary relationships is a well known property of neural networks. However, one of the main problems in using ANNs to provide support for therapy decisions is the need for a high level of trust in the predictions of such a model on the part of both the physician and the patient under examination. This need requires that a good generalization capability must be convincingly demonstrated. In a clinical context with a small data set, the key to good generalization lies in optimized complexity reduction techniques. Thus, improvement in these techniques will play an important role in increasing confidence in the application of ANNs to the clinical setting [48].

From earlier analysis on colon cancer, it is evident that FFANN enhanced diagnosis and prognosis when compared to other statistical methods, and increased survival prediction when compared to logistic regression or clinicopathological staging. However, the uncritical use of ANNs for prognostic and diagnostic classification of cancer, including colon cancer, can lead to the following mistakes: (1) the reported error rates for some ANNs may underestimate the true misclassification probabilities; for example, by not showing the cross validation error rates in the learning, validation and/or test sets, and in some cases by having a too small size of the test set; (2) fitting of biologically implausible functions to describe the probability of class membership when overfitting occurs, as overfitting generally occurs if the ratio between the number of observations and the number of parameters is smaller than two; (3) incorrectly and/or failure to report or describe the complexity of the network (i.e., number of parameters, the number of hidden layers and hidden units to calculate the number of fitted weights, etc.) will not allow the reader to judge the magnitude of overfitting, (4) use of inadequate statistical competitors or statistical methods to compare the performance of the networks. A fair comparison of the performance of FFNNs and statistical methods must be based on tools of similar flexibility like nearest-neighbor methods, generalized additive models, CART or logistic regression models with quadratic terms and multiplicative interaction terms, which is not usually carried out; (5) inefficient comparison with statistical methods without proving the significance of the differences between the observed misclassification rates, and (6) naive application of ANNs to survival data such as omitting censored cases (which lead to bias), and using the number of the time interval as an additional input unit, which causes the estimated survival probabilities not to depend on the length of the time intervals [45]. Avoiding the above mistakes when reporting the results of ANNs is a good science, as this will improve the confidence in the reliability of data reported in the scientific literature by using an unsupervised method such as ANN for data analysis, whose use has nevertheless been steadily on the rise?
